# A Novel Approach for the Detection and Genetic Analysis of Live Melanoma Circulating Tumor Cells

**DOI:** 10.1371/journal.pone.0123376

**Published:** 2015-03-25

**Authors:** Melody J. Xu, Mariana Cooke, David Steinmetz, Giorgos Karakousis, Deeksha Saxena, Edmund Bartlett, Xiaowei Xu, Stephen M. Hahn, Jay F. Dorsey, Gary D. Kao

**Affiliations:** 1 Department of Radiation Oncology, Perelman School of Medicine at the University of Pennsylvania, Philadelphia, Pennsylvania, United States of America; 2 Department of Surgery, Perelman School of Medicine at the University of Pennsylvania, Philadelphia, Pennsylvania, United States of America; 3 Department of Pathology, Perelman School of Medicine at the University of Pennsylvania, Philadelphia, Pennsylvania, United States of America; University of Queensland Diamantina Institute, AUSTRALIA

## Abstract

**Background:**

Circulating tumor cell (CTC) detection and genetic analysis may complement currently available disease assessments in patients with melanoma to improve risk stratification and monitoring. We therefore sought to establish the feasibility of a telomerase-based assay for detecting and isolating live melanoma CTCs.

**Methods:**

The telomerase-based CTC assay utilizes an adenoviral vector that, in the presence of elevated human telomerase activity, drives the amplification of green fluorescent protein. Tumor cells are then identified via an image processing system. The protocol was tested on melanoma cells in culture or spiked into control blood, and on samples from patients with metastatic melanoma. Genetic analysis of the isolated melanoma CTCs was then performed for BRAF mutation status.

**Results:**

The adenoviral vector was effective for all melanoma cell lines tested with sensitivity of 88.7% (95%CI 85.6-90.4%) and specificity of 99.9% (95%CI 99.8-99.9%). In a pilot trial of patients with metastatic disease, CTCs were identified in 9 of 10 patients, with a mean of 6.0 CTCs/mL. At a cutoff of 1.1 CTCs/mL, the telomerase-based assay exhibits test performance of 90.0% sensitivity and 91.7% specificity. BRAF mutation analysis of melanoma cells isolated from culture or spiked control blood, or from pilot patient samples was found to match the known BRAF mutation status of the cell lines and primary tumors.

**Conclusions:**

To our knowledge, this is the first report of a telomerase-based assay effective for detecting and isolating live melanoma CTCs. These promising findings support further studies, including towards integrating into the management of patients with melanoma receiving multimodality therapy.

## INTRODUCTION

Melanoma is the fifth most common solid malignancy in the United States, affecting 76,000 individuals each year [1]. Stage I disease has a 5-year survival rate of 92%, but survival drops precipitously for Stage II, III, and IV disease to 53%, 40%, and 15%, respectively [2]. New treatments have been recently developed, including targeted therapies and immune modulators in patients with advanced disease. Additional interest in combining immunomodulation and radiation therapy in patients with advanced melanoma have been fueled by the observation of abscopal effects, in which regression of metastatic cancer occurs distant from the irradiated site [3–5]. However, how best to monitor or stratify patients for different treatments or to detect early treatment failure remains unclear [6].

Circulating tumor cell (CTC) analysis may assist in the clinical management of melanoma. CTCs are cancer cells that have dissociated from the primary tumor and can be identified in peripheral blood through blood draws obtained with minimal risk [7]. CTCs are rare, usually representing no more than one in one million peripheral blood cells, and potentially carry prognostic significance, as suggested in studies of breast, colorectal, and prostate cancers [7–10]. Serial CTC counts before and after treatment may also help clarify disease status or risk of recurrence.

Because melanoma is derived from neural crest cells and thus often exhibits mesenchymal features, conventional CTC detection platforms designed for epithelial cancers using cell surface markers (such as epithelial cell adhesion molecule, EpCAM) may not be optimal for patients with melanoma. However, alternative cell surface markers, such as melanoma-specific cell surface proteoglycans, have aided the detection of CTCs in melanoma patients [11–15]. Clinical studies utilizing reverse transcriptase polymerase chain reaction (RT-PCR) to identify melanoma-specific RNA products in the blood have suggested potential prognostic value, though the precise origin of these products are unknown and may represent primary tumor mRNA shedding rather than CTC-derived mRNA [16–20]. Possible biological or technical hurdles with these CTC detection methods may include variability of cell surface marker expression or the uncertainty of the precise cellular origin of RT-PCR products, such as whether they are derived from live, dead, or dying cells.

We hypothesized that a novel telomerase-based assay not reliant on surface molecule expression would be effective for detecting melanoma CTCs. The assay relies on an adenoviral vector which expresses green fluorescent protein (GFP) driven by the human telomerase promoter in live cells. Telomerase is an enzyme that protects the ends of chromosomes to forestall senescence, and is upregulated in almost all tumor cells to help confer immortality [21,22]. In contrast, telomerase is downregulated in almost all normal cells, which are thus susceptible to senescence. This technique has been effective for a wide range of cancer cells and has successfully identified CTCs in patients undergoing radiation therapy for glioma, bladder cancer and non-small cell lung (NSCLC) cancer [23–27]. In this study, we tested the telomerase-based assay’s ability to identify melanoma cells in culture and CTCs in patients with melanoma, as well as its ability to enable genetic analysis of such cells, particularly mutant BRAF status.

## METHODS

### Cell Culture

Mel624, C8161, A375P, 451Lu melanoma cells were maintained in Roswell Park Memorial Institute medium (RPMI-1640, Invitrogen, Carlsbad, CA) supplemented with 10% fetal bovine serum (FBS, Invitrogen, Carlsbad, CA) and 1.0% penicillin-streptomycin at 37°C in an atmosphere of 5% CO_2,_ while MeWo, SK-Mel-2, and SK-Mel-5 cells were maintained in Eagle’s Minimal Essential Media (MEM, Mediatech, Inc, Manassas, VA) supplemented with 10% FBS (Invitrogen, Carlsbad, CA) and 1.0% penicillin-streptomycin at 37°C in an atmosphere of 5% CO_2_. U251 glioblastoma cells were grown in Dulbecco’s modified Eagle’s medium (DMEM, Invitrogen, Carlsbad, CA) with 10% FBS (Invitrogen, Carlsbad, CA) and 1.0% penicillin-streptomycin at 37°C in an atmosphere of 5% CO2. All cell lines were acquired from ATCC (Manassas, VA). Hoechst staining (Invitrogen, Carlsbad, CA) was used for live cell counting.

### Western blot analysis

Cells were harvested by scraping, centrifugation, and lysis on ice for 2 hours. Samples were then subject to electrophoresis, transferred to PVDF membranes and blocking against non-specific binding. The following antibodies were employed for specific protein detection: Pan-RAF (1:2000, mouse mAb, Cell Signaling Technology, Danvers, MA), BRAF^V600E^ (1:2000 mouse mAb, NewEast Biosciences, King of Prussia, PA), and β-actin (1:10,000, rabbit mAb, Cell Signaling Technology, Danvers, MA). Following incubation with the primary antibody, the membranes was rinsed with TBST, incubated for 5 minutes with horse radish peroxidase-conjugated secondary antibody, washed further with TBST, and finally developed with the Amersham Enhanced Chemiluminescence (ECL) kit or ECL Prime western blotting detection system (GE Healthcare, Little Chalfornt UK).

### Telomerase-based CTC Assay

The telomerase-based CTC assay utilizes a replication-competent adenovirus whose replication is regulated by the hTERT promoter element. A downstream CMV promoter drives GFP expression. Thus, a cell with increased telomerase activity produces increasing copies of the virus leading to amplified GFP expression detectable by fluorescence microscopy. The adenovirus is therefore employed as an agent to visually detect the presence of cancer cells in patient samples, i.e. as a “viral probe” to identify the presence of disease.

The CTC analysis protocol consists of the following: up to 10 mL of peripheral blood is collected in a sodium heparin tube, placed on ice, and processed within two hours of collection. The whole blood is combined with 10 mL of sterile phosphate-buffered saline (PBS) and introduced into an OncoQuick tube (Greiner Bio-One, Frickenhausen, Germany). After centrifugation of the OncoQuick tube, the buffy coat containing CTCs and the remaining WBCs are isolated and added to wash buffer (0.5% bovine serum albumin [BSA] in 1X PBS) for a second centrifugation step. The supernatant is discarded until 500 uL of solution remains. The remaining cells are re-suspended in 900 uL DMEM (Invitrogen, Carlsbad, CA) supplemented with 10% FBS (Invitrogen, Carlsbad, CA) and 1.0% penicillin-streptomycin. The cells are split evenly into two wells of an 8 well Poly-D-lysine chamber slide (Catalog #354632, Corning, Tewksbury MA) and incubated at 37°C in an atmosphere of 5% CO2. All samples are incubated with 2x10^8^ viral particles (total of 50 uL) to a total volume of 750 uL. At 24, 48, and 72 hours, the chamber slide is removed from the incubator and imaged using a computer-automated imaging program (Image Pro Plus 7.0, Media Cybernetics, Rockville, MD). The program can filter images by intensity, size, and other criteria to identify and enumerate GFP expressing cells.

### Determination of the adenoviral probe’s sensitivity and specificity for melanoma cells

Melanoma cell lines were exposed to the viral probe and imaged at 24, 48, and 72 hours to quantify GFP expression induced over time. Hoechst stain was added to cells prior to imaging to identify all nucleated cells on the plate. The GFP and Hoechst signal was enumerated for each well and sensitivity calculations were done for seven cell lines, with the experiment repeated four times. Healthy control blood was processed using the assay protocols and specificity determined by the ratio of false positive signal to total number of WBCs in each plate, with the study repeated twice for each of six healthy controls. Healthy volunteers provided written consent for blood draws for biomarker analysis according to University of Pennsylvania IRB approved protocol #814478.

Scatterplots displaying each detected cell by size and GFP intensity were obtained. By applying a rigorous GFP intensity threshold to exclude weak and background signals, false positives are able to be further excluded, thus maximizing specificity.

### Immunofluorescence staining

Melanoma cells grown *in vitro* were seeded on 8 well Poly-D-lysine chamber slides. Patient samples undergoing immunofluorescence staining were prepared according to the telomerase-based CTC assay protocol described above. Samples were fixed by media aspiration, PBS rinse followed by 4% formaldehyde fixation (15 minutes in room temperature), permeabilization with 0.25% Triton X-100 (10 minutes at room temp) followed by 2–3 washes with PBS, 5 minutes each. Samples were incubated with Melan-A antibody (1:50 mouse mAb, Santa Cruz Biotechnology, Dallas, TX) or BRAF^V600E^ antibody (1:200 mouse mAb, NewEast Biosciences, King of Prussia, PA). Samples were incubated with 1:200 chicken anti mouse-Alexa fluor (594) (Invitrogen, Grand Island, NY) for 1 hour at room temp. The cells were mounted with the media containing DAPI and then imaged using fluorescence microscopy. All the steps were followed by 3 washing steps with PBS, 5 minutes each.

### Genetic analyses of isolated cancer cells

The selected isolation of cells was performed using a capillary-based vacuum-assisted cell acquisition system (Kuiqpick, NeuroInDx; S1 Fig.) [28]. Calibration was initially performed under bright field conditions, followed by collection (via micro-capillary aspiration) under fluorescence microscopy of individual cancer cells rendered fluorescent after exposure to the probe. Total genomic DNA was extracted from fluorescent tumor cells that had been collected and pooled (1–10 cells) and subsequently amplified using Single Cell Whole Genome Amplification Kit (Yikon Genomics Co. Ltd, Taizhou, China) [29]. After genomic DNA amplification, PCR products were purified using Agencourt AMPure XP kit (Beckman Coulter, Brea, CA). Amplified and purified genomic DNA from collected cells were then analyzed using eQ-PCR BRAF Detection Kit (TrimGen Corporation, Sparks, MD) and subsequently sequenced. As additional confirmation, the purified DNA was also analyzed for BRAF mutations via the Mutector kit (TrimGen Corporation, Sparks, MD, USA) which is based on the Shifted Termination Assay (STA) [30,31]. The STA contains reagents that allow for detection of not only the V600E mutation, but also our other mutations affecting this amino acid (V600K, V600R, V600A, and V600G). In each case, eQ-PCR and Mutector results were consistent, detecting only the V600E mutation. Experiments with cancer cells in culture, spiked control blood (from healthy volunteers) and melanoma patients were all repeated at least twice, with repeated confirmatory qPCR reactions performed as well for additional confidence.

### Pilot study and control blood samples

In partnership with the Abramson Cancer Center, which maintains a biobank protocol approved by the University of Pennsylvania Institutional Review Board (Protocol #703001), blood samples were collected by clinical research coordinators after written informed consent was obtained from eligible patients with metastatic melanoma. The protocol provides for research blood samples that are de-identified and distributed to requesting laboratories for biomarker analysis, including for this study. After completion of sample processing, clinical research coordinators provided de-identified patient demographics and disease course details for correlative statistical analysis. The median age of the patients included in this study, was 63 (range 38–71) and 70% of the patients were female.

For control blood, healthy volunteers without prior history of cancer and between ages 18–60 gave consent for blood draws of up to 20 mL on an IRB-approved control blood study. For cell spiking experiments, 500 cells were counted using Nexcelom Biosciences’ Cellometer (Lawrence, MA) and then added to the blood sample prior to sample processing. Spiked experiments were done for four melanoma cell lines, and repeated three times. The recovery rate for spiked control blood experiments (20–50%) was calculated by dividing the number of GFP+ cells recovered by the total number of spiked cells, and potentially affected by factors such as cell death, mechanical shear forces, or adhesion to matrix or plastic. Importantly, cellular proliferation is not a confounding factor in spiked control blood experiments because the adenoviral infection inhibits cell division.

### Statistics

Statistical analysis was done with the STATA program (StataCorp, College Station, TX). Descriptive statistics were used for sensitivity and specificity analysis. ANOVA and linear regression models were used to analyze CTC counts with patient data from the pilot study. Univariate and multivariate analysis was done to establish associations between binary variables (e.g. sex, BRAF WT vs mutant) and CTC counts as a continuous outcome variable. ROC curves were generated using data from blood samples of healthy volunteers and patients with known melanoma [32].

## RESULTS

### Melanoma cells in culture and in patients are identified by the telomerase-detecting viral probe

**Fig 1:**
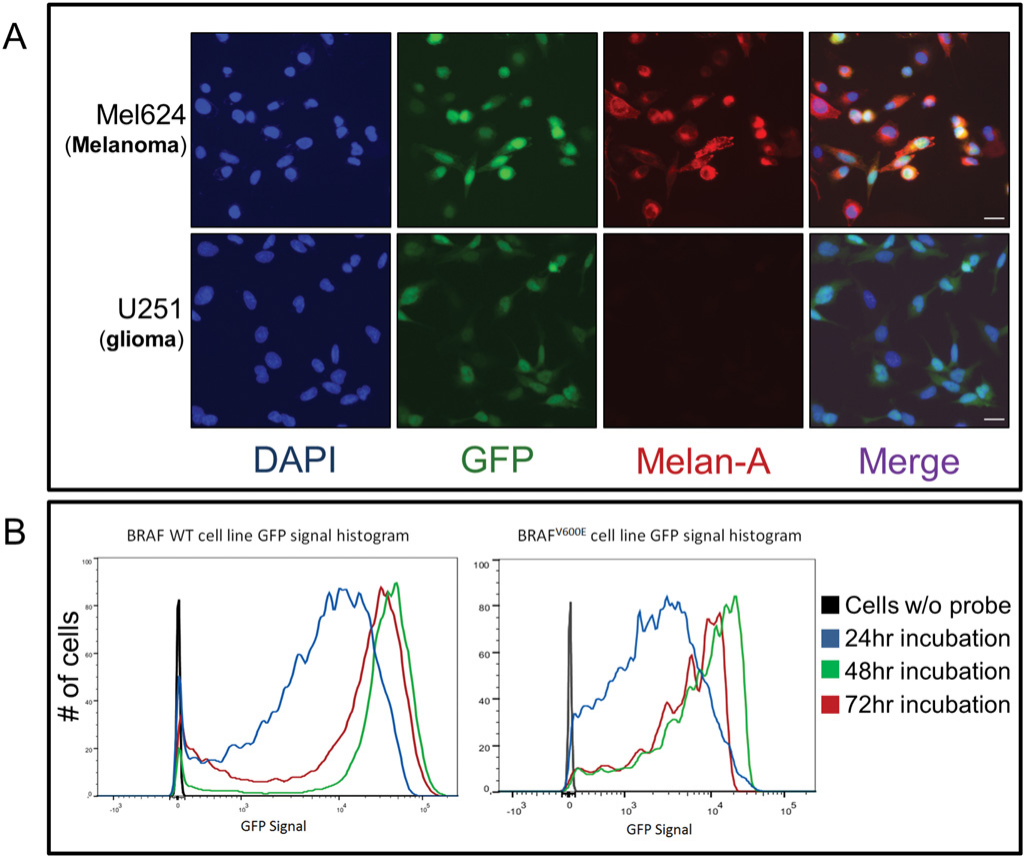
Pre-clinical characterization of the viral probe’s efficacy in melanoma cells. (A) Melanoma and glioma cells (Mel624 and U251, respectively, are shown in the top and bottom rows as representative examples) were incubated with the probe, followed by immunofluorescence staining for Melan-A. Robust GFP expression indicated the efficacy of the probe for melanoma was comparable to that for glioma. The identity of melanoma cells is confirmed by Melan-A expression that coincided with GFP. Each row shows the same cells, with DAPI as a nuclear stain in the left-most panel, and the rightmost panel (Merge) show merged images of all three fluorescent channels. Bar, 30 um. (B) Flow cytometry analysis of BRAF WT (MeWo) and BRAF mutated (Mel624) cells indicated that for both cell lines, GFP signal peaked after 48 hours of exposure to the viral probe, with no further increase after 72 hours.

We previously found that melanoma human telomerase reverse transcriptase (hTERT) levels were comparable to other tumors in which the probe has been found to be effective, including glioma and NSCLC cells [26,27]. These elevated hTERT levels are consistent with previous reports of elevated telomerase levels in melanoma, thus supporting the potential usefulness of a viral probe that detects high levels of telomerase activity in melanoma CTCs [21,22]. The high levels of telomerase activity in melanoma cells translated to strong expression of fluorescence after exposure to the viral probe and did not interfere with Melan-A expression (Fig. 1A). We were further encouraged that the probe resulted in strong fluorescence in melanoma cells regardless of BRAF wild type (WT, left panel) or mutant (right panel) status, and with peak fluorescence reached by 48 hours (Fig. 1B). The probe was found to be very effective in all melanoma cell lines tested, including those of different genetic backgrounds (average sensitivity of 88.7% [95%CI 85.6–90.4%], S2B Fig.), with efficacy comparable to cell lines derived from other tumors for which the viral probe has been found to be effective, including glioma, non-small cell lung cancer, and bladder carcinoma [25–27].

**Fig 2:**
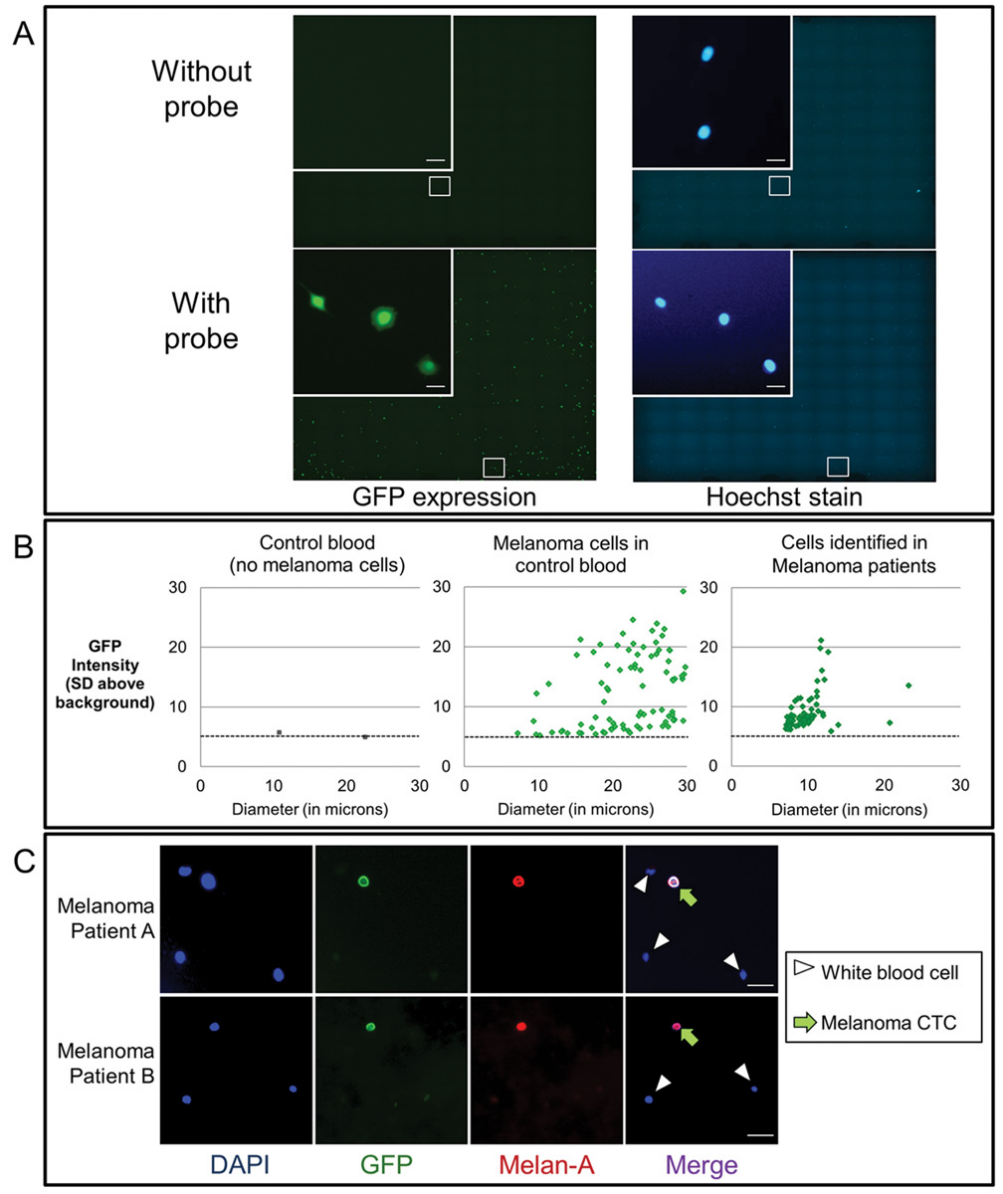
Integration of the viral probe with a semi-automated, computer-driven image acquisition and analysis system. (A) Melanoma cells (A375P cells shown here as a representative example) incubated with the probe for 24 hours were visualized under fluorescent microscopy. Tiled images were taken for each well. Hoechst dye was added before image acquisition to delineate nucleated cells, which take up the dye and emit blue fluorescent signal under UV light. Bar, 30um. (B) Scatter plots showing individual cells, plotting size (X-axis) and fluorescent intensity (standard deviations (SD) above background) (Y-axis) identified by the imaging program. Control blood studies from a single subject with spiked melanoma cells (middle graph) or without spiked cells (left graph) demonstrated that a stringent GFP intensity cutoff (black dotted line) excluded false positive signals and contributed to greater specificity for patient samples. The size and fluorescent intensify cutoffs were applied to the blood samples of patients with metastatic melanoma (data from one representative patient is shown in the right graph). Color choices are for aesthetic purposes only. Melanoma cells in culture (middle panel, showing 60% of the total cells identified by the imaging program, not meant to reflect recovery rate of spiked melanoma cells) rapidly adhere to the culture surface and hence show greater variability in size compared to the CTCs from patients with melanoma (right panel). (C) Confirmation of melanoma origin of CTCs. Patient samples were fixed after exposure to probe, and immunofluorescence then performed for Melan-A and DAPI. The DAPI+/GFP+/Melan-A+ (arrows) cells confirm the identification of melanoma CTCs while the adjacent DAPI+ only cells (arrowheads) consist of white blood cells. Bar, 30 um.

These encouraging results enabled the integration of the viral probe with a semi-automated, computer-driven image acquisition and analysis system (Fig. 2A, S2A Fig.). This system has been described in previous publications and incorporates reproducible cell identification and imaging, as well as filters for size and fluorescence, so that that cellular debris and cells with weak fluorescence are excluded from analysis [25–27]. As an additional validation step prior to testing patient samples, we performed control experiments in which peripheral blood samples from healthy volunteers were analyzed alone or spiked with melanoma cells, and in each case exposed to the probe. In the control blood without melanoma cells, the viral probe resulted in almost no fluorescence (specificity of 99.9% [95%CI 99.8%-99.9%]) (Fig. 2B, left panel). In contrast, robust fluorescence was detected in the control blood spiked with melanoma cells (Fig. 2B, middle panel).

### Pilot study of the telomerase-based assay in patients with Metastatic Melanoma

Having validated the telomerase-based assay for the detection of melanoma cells, we initiated a pilot study in patients with metastatic melanoma. After exposure to the viral probe, patient samples were imaged and individual melanoma cells were readily identified by software analysis (Fig. 2B, right panel). Interestingly, the melanoma cells in patient samples showed a range of fluorescence that was comparable to that of cells in culture, but less variable in size. This may reflect that cells in culture have been selected for hypermutability and aggressive growth characteristics, including rapid adhesion to cell culture surfaces. The latter results in much larger cellular diameters, as cells in culture attach and spread out on the surface of the slides. Nonetheless, as an additional confirmatory step, counterstaining for Melan-A was performed on the patient blood samples after initial analysis. Expression of Melan-A coincided with the GFP in melanoma cells, while white blood cells showed neither GFP nor Melan-A, collectively indicating high specificity for the telomerase-based CTC assay (Fig. 2C).

**Fig 3:**
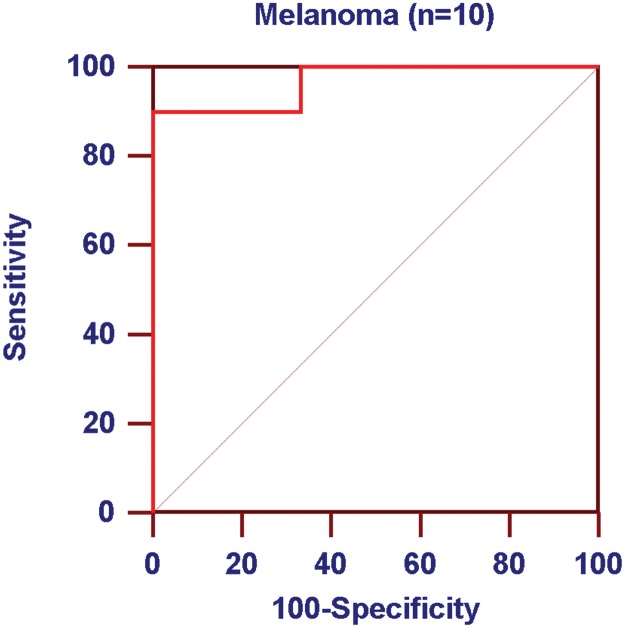
ROC curve demonstrating favorable telomerase-based CTC assay test characteristics for identifying patients with and without melanoma. A CTC count threshold of 1.1 CTCs/mL results in sensitivity of 90.0% and specificity of 91.7%. Analysis was performed using blood samples from 10 melanoma patients and 6 controls.

Receiver operator characteristic (ROC) curves were generated using cell counts from the pilot (n = 10) and control blood (n = 6) studies. This indicated that a CTC count threshold of 1.1 CTCs/mL was able to correctly detect metastatic melanoma with a sensitivity of 90.0% and specificity of 91.7% (Fig. 3). This demonstrates that the telomerase-based assay showed desirable test characteristics because not only are detected cells viable and potently fluorescent, they are also detected in sufficient numbers so as to delineate diseased individuals from healthy controls with high sensitivity and specificity.

Descriptions of the patients in the pilot study, listed in order of ascending CTC counts, are shown in Table 1. The majority of subjects had dermal, lymph node, or recurrent skin disease and four had visceral involvement (brain, lung, gallbladder, bowel, etc.) prior to treatment. Four subjects were considered to have low burden of disease (only one involved organ) but high risk of recurrence. Three subjects were considered to be without radiographic evidence of disease (no radiographic evidence of disease, NED) despite having diagnoses of metastatic melanoma. For example, a 49 year old man first underwent resection of melanoma metastases in the small bowel, lung, and brain and had no further progression or new sites of disease for the next 12 years, including when the CTC analysis was performed during follow-up. Now 61 years old, all diagnostic imaging shows no evidence of disease so is classified as “NED” in Table 1. The blood samples from nine of the ten patients contained detectable CTCs, with a median CTC count of 2.7 CTCs/mL and a range from 0.7 to 27.1 CTCs/mL.

**Table 1:** Pilot study patient demographics, disease course characteristics at time of the CTC analysis.

CTCs/mL	Age	Sex	Site of Metastases	Initial Disease Burden	BRAF mutation status	History of Chemo-therapy	History of Immuno- therapy	6 Mo. Disease Status
0.7	60	F	Gingiva	NED	WT	N	N	Stable
1.2	66	M	Subcutaneous skin and distant LN	High	WT	Y	Y	PD
1.4	71	F	Bone and gallbladder	High	WT	Y	Y	Stable
1.8	70	F	In-transit skin	Low	WT	N	Y	Stable
2.0	65	F	Thigh	Low	WT	Y	Y	PD
3.4	61	M	Lung, brain, and small bowel	NED	WT	N	N	Stable
4.3	61	F	Face	NED	Mutant	N	Y	Stable
6.6	53	F	Scalp	Low	WT	N	Y	PD
11.5	66	F	Lung	Low	WT	N	Y	PD
27.1	38	M	Lung, neck, and LN	High	Mutant	N	Y	PD

Nine of ten patients had detectable CTC counts (defined via the ROC curve analysis as greater than 1.1 CTCs/mL). NED = no radiographic evidence of disease, LN = lymph node involvement, WT = wild type, PD = recurrent or progressive disease.

In univariate analysis of the pilot patient results, only the presence of a BRAF mutation showed a trend toward significant correlation with increased CTC levels (p = 0.051). In contrast, none of the other factors significantly correlated with increased CTC levels (including age, sex, burden of disease, site of metastasis, and recent history of chemo- or immune therapy) (Table 2).

**Table 2:** Results of one-way analysis of variance for single variables and linear regression model for multivariate analysis.

Univariate analysis	p-value	
Age (<65 vs ≥65)	0.38	
Sex	0.27	
Site of metastasis (dermal or LN vs other viscera)	0.13	
Burden of disease (NED vs low vs high)	0.31	
BRAF mutation status (WT vs mutant)	0.051	
History of chemotherapy	0.28	
History of immune therapy	0.47	
**Multivariate analysis**	**Regression Coefficient**	**p-value**
Burden of disease (NED vs low vs high)	7.20	0.03
BRAF mutation status (WT vs mutant)	8.02	0.10
History of chemotherapy	-10.94	0.04

In the univariate analysis, all factors were not significantly associated with observed CTC levels, although there was a trend toward significance (p = 0.051) between the association between higher counts and the presence of the BRAF mutation. In the multivariate analysis, burden of disease and history of chemotherapy were significantly correlated to CTC levels (p = 0.03 and p = 0.04, respectively). NED = no evidence of disease, LN = lymph node, WT = wild type.

A linear regression model was then constructed, taking into consideration burden of disease, BRAF mutation status, and history of chemotherapy treatment. This multivariate analysis revealed that higher CTC counts were significantly correlated with greater burden of disease (p = 0.03), while, in contrast, lower CTC counts significantly correlated with recent history of cytotoxic chemotherapy (p = 0.04). By this multivariate analysis, BRAF mutation status was no longer found to be significantly associated with higher CTC levels, probably due to the greater influence of disease burden or effects of treatment.

The results of CTC assays may reflect the extent of disease or responsiveness to treatment, but might also have prognostic value. To investigate the possibility of the latter, we reviewed the clinical status of each patient at six months after the CTC analysis was performed, with the results as shown in the last column of Table 1. Intriguingly, if the CTC level was ≥2 CTCs/mL, the odds ratio (OR) of developing recurrence or progression of disease at six months was 6.0 (95%CI 0.4–101.6, p = 0.21). Although the sample size is small and the interval between the CTC analysis and follow-up substantial, these results suggest potential usefulness (80.0% sensitivity and 60.0% specificity) for predicting progression of disease with the telomerase-based assay. This may merit a larger study better powered for statistical significance and designed to include sequential analysis, which enable the detection of increased CTCs for patient with progressive disease and unchanged or decreased CTCs in patients with stable disease.

### The telomerase-based assay allows isolation of individual Melanoma Circulating Tumor Cells and characterization of their BRAF mutation status

**Fig 4:**
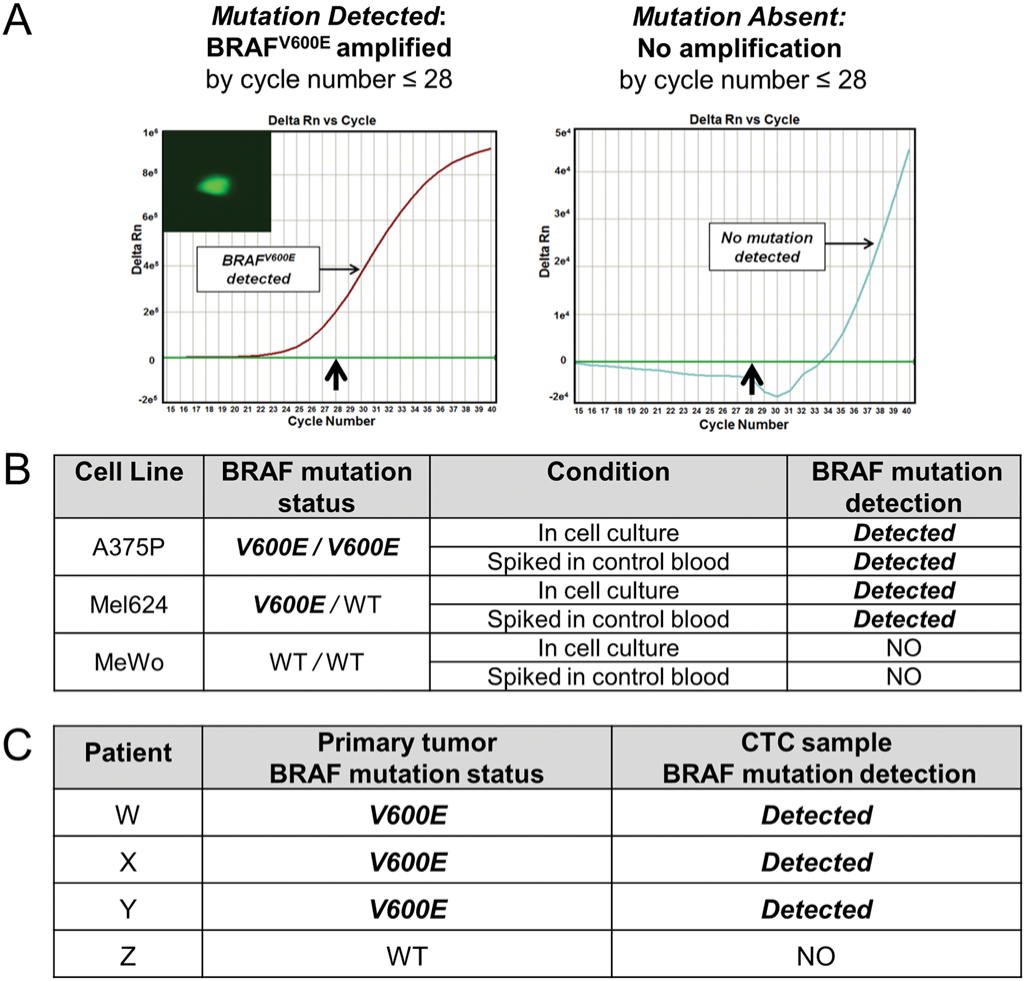
Detection of BRAF mutations in isolated melanoma cells in culture and in CTCs from melanoma patients. (A) PCR results indicating the presence or absence of the BRAF^V600E^ mutation. Cells exposed to the probe and rendered fluorescent (left inset) were isolated via capillary-based methods. Whole genome amplification (WGA) was performed on the DNA extracted from each cell, followed by quantitative polymerase chain reaction (qPCR) analysis using primers specific for the BRAF^V600E^ mutation. The presence of the mutation results in signal (Delta Rn, Y-axis) detectable by the 28th cycle and a curve of the characteristic “s” shape (left, arrow points to 28th cycle). The absence of the mutation results in no characteristic curve by the 28th cycle (right, arrow points to 28th cycle). (B) Isolation and genetic analysis for mutated BRAF^V600E^ in melanoma cells in culture and spiked in control blood. A375P (homozygous BRAF^V600E^ mutated), Mel624 (heterozygous BRAF^V600E^ mutated), and MeWo (homozygous BRAF WT) cells were isolated in culture and after being spiked into control blood using the capillary-based technique described. The DNA was extracted from each cell and subject to WGA, followed by qPCR analysis with primers specific for the BRAF^V600E^ mutation. In each case, the qPCR analysis accurately confirms the presence or absence of the BRAF^V600E^ mutation for each cell line. (C) Isolation of CTCs from patients and subsequent genetic analysis for BRAF mutation status. CTCs were isolated from an additional cohort of patients via capillary-based methods followed by DNA extraction, WGA, and qPCR analysis for BRAF^V600E^. In each case, the BRAF mutation status of the isolated CTCs corresponded to that of the primary tumor.

We then investigated whether identified CTCs could be isolated and subject to additional analyses, particularly analyses for specific genetic mutations of therapeutic relevance for melanoma. We therefore developed protocols utilizing capillary-based isolation of individual cells (manuscript in preparation, Fig. 4, S1 Fig. and S4A Fig.). We first tested melanoma cells in culture, characterizing A375P (homozygous BRAF^V600E^), Mel624 (heterozygous BRAF^V600E^), and MeWo (homozygous BRAF WT) cell lines for BRAF protein expression (Pan-RAF and mutant BRAF^V600E^) on western blotting and immunofluorescence (S3 Fig.). Each of these cell lines was then exposed to the viral probe, followed by micro-capillary aspiration of the resultant fluorescent cells, from which DNA was extracted. Whole genome amplification (WGA) was performed on the extracted DNA, followed by quantitative polymerase-chain reaction (qPCR) for the BRAF^V600E^ mutation (Fig. 4A, S3C Fig., and S4 Fig.). These experiments indicated that the exposure to the viral probe and the resultant GFP expression did not interfere with the ability to extract DNA and successfully perform WGA of the DNA. Furthermore, the BRAF^V600E^ mutation was preserved in the DNA after WGA and could be readily detected via qPCR. As an additional confirmatory step, a second method of BRAF mutation analysis was performed that was capable of determining different BRAF V600 mutations. The STA-based method, as described in the Methods and Materials section, confirmed that the identified BRAF mutation was exclusively the V600E mutation and not V600K, V600R, V600A, or V600G.

As a final step before testing in clinical samples, these experiments were repeated with each of the cell lines spiked into blood from healthy volunteers. Similar to the experiments described previously, individual cells were isolated, pooled and extracted for DNA which then underwent WGA, and then subject to qPCR analysis for BRAF status. These results indicated that the BRAF mutation status of tumor cells in blood samples could be accurately determined using the methods developed (Fig. 4B, S4C Fig., and S5C Fig.).

Encouraged by the success of these experiments, we tested the protocol on blood samples from an additional cohort of patients (Fig. 4C, S4D Fig., and S5D Fig.). For each of the patients, the telomerase-based assay identified CTCs, which were then isolated via the same microcapillary-based procedures, pooled, extracted for DNA, and then underwent WGA. For each of the patients, the results of the qPCR analysis for BRAF mutation in the DNA extracted from CTCs matched the BRAF mutation status of the primary tumor.

## DISCUSSION

To our knowledge, this study is the first to describe a telomerase-based approach to detecting melanoma CTCs. We aimed to demonstrate the feasibility of this CTC assay through pre-clinical studies and in a pilot study of melanoma patients. The adenoviral probe was found to be highly sensitive (88.7%) and specific (99.9%) for melanoma cells and its efficacy was not affected by BRAF mutation status. The melanoma-origin of the detected cells in culture and in samples from patients was confirmed via co-staining for anti-Melan-A, thus distinguishing melanoma cells (DAPI+/GFP+/Melan A+) from the surrounding WBCs (DAPI+ /GFP- /Melan A-) (Fig. 2C). The GFP expression in cancer cells exposed to the viral probe enabled analysis and optimization via flow cytometry techniques and quantification via semi-automated computer image analysis. Finally, we applied the protocol to patients with metastatic melanoma, which successfully detected CTCs in the majority of patients tested. These results enabled the calculation of ROC curve, indicating favorable test characteristics whereby the telomerase-based assay is able to identify patients with and without melanoma with high sensitivity and specificity (90.0% and 91.7%, respectively).

A number of observations merit comment. In the multivariate analysis, increased burden of disease was found to be associated with increased CTC levels (p = 0.03). This appears reasonable, as more aggressive diseases may be associated with areas of increased or leaky vasculature, or may be expected to seed more CTCs or micrometastases into the bloodstream. On the other hand, history of recent cytotoxic chemotherapy was associated with decreased CTC levels (p = 0.04). This may be due to systemic chemotherapy clearing CTCs from the circulation, or the diminished ability of dead or dying cancer cells to express fluorescence (an effect we have noted with *in vitro* experiments). Additional studies would be needed to determine the ideal interval after chemotherapy to perform the assay and/or if the assay’s ability to detect live cancer cells and exclude dead or dying cells proves advantageous for determining prognosis. Detection of CTCs was achieved in patients with either WT or mutant BRAF melanoma. In the univariate analysis, BRAF mutation showed a trend toward association with higher CTC counts (p = 0.051), possibly reflecting the biological aggressiveness of mutant BRAF disease [33,34]. The association however was not significant in multivariate analysis, likely due to the larger influence of other factors such as increased burden of disease.

CTC detection and isolation techniques are useful not only because the number of CTCs may mirror the burden or clinical aggressiveness of the existing disease, but also because they can potentially supply biological material from the tumor with no additional risk to patients. Towards the latter, we have established microcapillary-based procedures that enable the collection of melanoma CTCs rendered fluorescent by the telomerase-based assay. We have further shown that DNA extracted and amplified from the collected CTCs enable BRAF^V600E^ mutational analysis, the result of which match the BRAF status of the originating tumor. This potentially paves the way for sequential analyses of disease status in patients undergoing BRAF-targeting agents, and may complement or represent an advance over previous efforts at tracking genetic changes in melanoma [14,35].

Other potential strengths of this telomerase-based assay include:

1. Live cell detection. As live cells are required for probe infection and production of GFP, this technique ensures that all detected CTCs are live. Compared to cells identified by surface marker expression alone, which can be either live or dead at detection, cells detected using this telomerase-based assay may have greater biological significance and metastatic potential, thus the trending of these cells may have implications toward the development of metastatic disease or may be more closely associated with response to treatment.
2. Versatile and reliable quantification. The GFP signal resulting from the probe can be easily quantified using both flow cytometry and fluorescent microscopy (including coupling with semi-automated computer image processing). This enables the establishment of parameters distinguishing CTCs from background noise on the basis of fluorescent intensity or size.
3. Potential integration with existing CTC detection assays. One can envision adapting this probe to direct cancer cells to express other reporter proteins including other fluorescent proteins, enabling integration into different imaging or enrichment modalities and existing CTC analysis platforms. This telomerase-based assay may also complement techniques which focus on the serum component of peripheral blood draws, as it only requires isolation of the mononuclear layer and would not impede simultaneous analysis of circulating DNA or mRNA.

The telomerase-based CTC assay has potential limitations. It is possible that cancer cells in culture have been selected for high protein expression and aggressive growth characteristics, different from the unselected circulating melanoma cells within a patient’s circulation. Telomerase may not be highly expressed in all tumor cells, with alternate pathways possibly in effect to protect telomeres, such as alternative lengthening of telomeres, or ALT, phenotypes, as described in approximately 7% of malignant melanomas [36]. However, we have yet to find a cultured cancer cell line for which this approach has not been effective, or solid tumor patient populations for which the assay has not shown efficacy [25–27]. Nonetheless, there remains another theoretical possibility that the live cell requirement of the telomerase-based assay may miss cancer cells that are not dead or dying but temporarily dormant and which may be reactivated under exceptional circumstances.

A more practical limitation may be that attention must be devoted for logistical considerations after the sample has been collected from the patient. The live cell requirement of the CTC assay requires that variations in temperature or time in transit be minimized, and the processing of the samples requires a setup that can accommodate handling of adenoviruses. The need for incubation of the patient sample with the adenoviral probe for 48 hours places further demands on staff time and resources. Thus, the telomerase-based assay would likely require rapid climate-controlled access to a centralized laboratory setting with ample, flexible and dedicated staffing.

## CONCLUSION

This is the first report to our knowledge of a telomerase-based method for detecting and isolating melanoma CTCs. This assay was effective in identifying melanoma cells in culture with high sensitivity and specificity. In a pilot study of patients with metastatic melanoma, the telomerase-based assay was effective in detecting CTCs in almost all patients. We also report proof-of-principle data demonstrating the feasibility of a novel microcapillary-based approach for isolating individual CTCs from patient samples, whereby the CTC BRAF status matched that of the originating tumor. Together, these observations support the potential usefulness of the telomerase-based CTC assay for patients with melanoma.

## Supporting Information

S1 Fig NeuroInDx Kuiqpick system. *In vitro* validation of capillary-based isolation of GFP probe-expressing cancer cells. Pre-collection and post-collection imaging of a target cell expressing GFP that is removed by capillary-based isolation (red circle). Disposable capillary unit (DCU) imaging (under phase contrast and fluorescence (GFP) conditions) demonstrates the capture and isolation of a single cell.
(TIF)

S2 Fig Determination of viral probe sensitivity for various melanoma cell lines. (A) Representative scatter plots showing individual cells, plotting size (X-axis) and fluorescent intensity (standard deviations (SD) above background) (Y-axis) identified by the imaging program. Seven melanoma cell lines were incubated with the probe and GFP+ cells were enumerated using size and fluorescence intensity criteria described in Methods. Hoechst staining was used to detect the number of nuclei in the well. Data for five cell lines shown: A375P (380 GFP+/428 nuclei), Mel624 (400 GFP+/417 nuclei), MeWo (256 GFP+/267 nuclei). SK-Mel-2 (307 GFP+/348 nuclei), and C8161 (271 GFP+/287 nuclei). Colors are for aesthetic purposes only. (B) Table describing each cell line’s clinical origin, BRAF mutation status, and sensitivity to the probe with 95% CI. Sensitivity calculated by dividing # GFP+ cells by # Hoechst-stained nuclei. 95% CI calculated by 4–6 repeated experiments for each cell line. The C8161 melanoma cell line is BRAF G464E mutant and wild type at the 600 codon [37,38].
(TIF)

S3 Fig Characterization of the BRAF status of melanoma cell lines. (A) Western blotting showing that BRAF protein (“Pan-RAF”, top blot) is present in all cell lines. However, probing with an antibody specific for the mutated BRAF protein (“BRAF^V600E^”, middle blot) reveal that only A375P and Mel624 express the mutated protein. This data is consistent with sequencing results for each cell line as well as the subsequent WGA and qPCR analysis. Probing for β-actin served as a loading control. NSCLC = non-small cell lung cancer. (B) Immunofluorescence staining of A375P (homozygous BRAF^V600E^) and MeWo (homozygous BRAF WT) cell lines with DAPI and the BRAF^V600E^ antibody, are consistent with the western blot and sequencing results. Bar, 30 um. (C) The A375P cell line was incubated with the probe and DNA was extracted, amplified, and subject to qPCR analysis for the BRAF allele. PCR results demonstrated amplification of the BRAF^V600E^ allele and absence of amplification of the BRAF WT allele.
(TIF)

S4 Fig Detection of BRAF^V600E^ DNA in isolated melanoma cells in culture, spiked into control blood, and CTCs from patients with melanoma. (A) Isolation, processing, and analysis of individual cells. Cells exposed to the probe and rendered fluorescent were isolated via capillary-based methods. The individual cells within the glass capillary tubes can be visualized under bright field (left) and fluorescence microscopy (right). Whole genome amplification (WGA) was performed on the DNA extracted from each cell, followed by quantitative polymerase chain reaction (qPCR) analysis using primers specific for the BRAF^V600E^ mutation. The presence of the mutation results in signal (Delta Rn, Y-axis) detectable by the 28th cycle and a curve of the characteristic shape (as shown in the graph resulting from the BRAF^V600E^ kit control). Bar, 30 um. (B) Isolation and genetic analysis of melanoma cells in culture. A375P (homozygous BRAF^V600E^), Mel624 (heterozygous BRAF^V600E^), and MeWo (homozygous BRAF WT) cells were isolated using the capillary-based technique described. The DNA was extracted from each cell and subject to WGA, followed by qPCR analysis with primers specific for the BRAF^V600E^ mutation. Inset images show representative isolated cells. In each case, the qPCR analysis confirms the specific BRAF status of the parental cells in culture. (C) Isolation and genetic analysis of melanoma cells spiked into control blood. Melanoma cells were prepared as in (B) but spiked into blood from healthy volunteers. The subsequent isolation, DNA extraction, WGA, and qPCR analysis for BRAF mutations was not impeded by the presence of blood, and again the results matched that of the original cells. (D) Isolation of CTCs from patients and subsequent genetic analysis for BRAF mutation status. These methods described above were was applied to blood samples from an additional cohort of patients, with CTCs isolated via capillary-based methods followed by DNA extraction, WGA, and qPCR analysis for BRAF. In each case, the BRAF mutation status of the isolated CTC corresponded to that of the primary tumor. qPCR amplification curves demonstrating strong amplification of the BRAF^V600E^ allele in Patients W and Y, who were found to have mutated BRAF in the primary tumor. qPCR amplification curve of patient Z corresponds to the primary tumor’s BRAF WT mutation status.
(TIF)

S5 Fig Detection of BRAF WT DNA in cells in culture and spiked into control blood. Each isolated melanoma cell analyzed for the BRAF^V600E^ mutation qPCR analysis also underwent qPCR analysis using primers specific for BRAF WT. (A) The presence of the WT allele results in signal (Delta Rn, Y-axis) detectable by the 28th cycle (as shown in the graph resulting from the BRAF WT kit control). (B) A375P (homozygous BRAF^V600E^), Mel624 (heterozygous BRAF^V600E^), and MeWo (homozygous BRAF WT) cells were exposed to the probe, followed by capillary-based isolation, WGA, and qPCR analysis. In each case, the results of the qPCR analysis confirm the specific BRAF allele status of the parental cells in culture. (C) Isolation and genetic analysis of melanoma cells spiked into control blood. Melanoma cells were prepared as in (B) but spiked into blood from healthy volunteers. The qPCR analysis demonstrated non-melanoma-specific BRAF WT amplification in each case. This is likely a result of WBCs co-isolating with cancer cells under the capillary-based cell collection protocol. (D) Isolation of CTCs from patients and subsequent genetic analysis for BRAF mutation status. The qPCR analysis also demonstrated non-melanoma-specific BRAF WT amplification in each case. As in the case of melanoma cells spiked into control blood, the presence of BRAF WT DNA is likely due to normal WBCs co-isolating along with the cancer cells, and thus the WBC DNA also being co-amplified during WGA.
(TIF)

## References

[1] U.S. Cancer Statistics Working Group. United States cancer statistics: 1999–2008 Incidence and mortality web-based report. Atlanta: U.S. Department of Health and Human Services, Centers for Disease Control and Prevention and National Cancer Institute; 2013 Available: www.cdc.gov/uscs.

[2] American Cancer Society. What are the survival rates for melanoma skin cancer by stage? Available: http://www.cancer.org/cancer/skincancer-melanoma/detailedguide/melanoma-skin-cancer-survival-rates. Accessed 2013 Jul 16.

[3] Postow MA, Callahan MK, Barker CA, Yamada Y, Yuan J, Kitano S, et al Immunologic correlates of the abscopal effect in a patient with melanoma. N Engl J Med. 2012;366: 925–931. 10.1056/NEJMoa1112824 22397654 PMC3345206

[4] Stamell EF, Wolchok JD, Gnjatic S, Lee NY, Brownell I. The abscopal effect associated with a systemic anti-melanoma immune response. Int J Radiat Oncol Biol Phys. 2013;85: 293–295. 10.1016/j.ijrobp.2012.03.017 22560555 PMC3415596

[5] Okwan-Duodu D, Pollack BP, Lawson D, Khan MK. Role of radiation therapy as immune activator in the era of modern immunotherapy for metastatic malignant melanoma. American Journal of Clinical Oncology. 2013 [Epub ahead of print] 10.1097/COC.0b013e3182940dc3 23648438

[6] Jarkowski A 3rd, Norris L, Trinh VA. Controversies in the management of advanced melanoma: "Gray" areas amid the "black and blue". Ann Pharmacother. 2014; 48(11):1456–68. 10.1177/1060028014544165 25056920

[7] Paterlini-Brechot P, Benali NL. Circulating tumor cells (CTC) detection: Clinical impact and future directions. Cancer Lett. 2007;253: 180–204. 17314005 10.1016/j.canlet.2006.12.014

[8] Steen S, Nemunaitis J, Fisher T, Kuhn J. Circulating tumor cells in melanoma: A review of the literature and description of a novel technique. Proc (Bayl Univ Med Cent). 2008;21: 127–132. 18382750 10.1080/08998280.2008.11928377 PMC2277345

[9] Cristofanilli M, Budd GT, Ellis MJ, Stopeck A, Matera J, Miller MC, et al Circulating tumor cells, disease progression, and survival in metastatic breast cancer. N Engl J Med. 2004;351: 781–791. 15317891 10.1056/NEJMoa040766

[10] Pantel K, Brakenhoff RH, Brandt B. Detection, clinical relevance and specific biological properties of disseminating tumour cells. Nat Rev Cancer. 2008;8: 329–340. 10.1038/nrc2375 18404148

[11] Rao C, Bui T, Connelly M, Doyle G, Karydis I, Middleton MR, et al Circulating melanoma cells and survival in metastatic melanoma. Int J Oncol. 2011;38: 755–760. 10.3892/ijo.2011.896 21206975

[12] Khoja L, Lorigan P, Zhou C, Lancashire M, Booth J, Cummings J, et al Biomarker utility of circulating tumor cells in metastatic cutaneous melanoma. J Invest Dermatol. 2013;133: 1582–1590. 10.1038/jid.2012.468 23223143

[13] Karakousis G, Yang R, Xu X. Circulating melanoma cells as a predictive biomarker. J Invest Dermatol. 2013;133: 1460–1462. 10.1038/jid.2013.34 23673501

[14] Sakaizawa K, Goto Y, Kiniwa Y, Uchiyama A, Harada K, Shimada S, et al Mutation analysis of BRAF and KIT in circulating melanoma cells at the single cell level. Br J Cancer. 2012;106: 939–946. 10.1038/bjc.2012.12 22281663 PMC3305957

[15] Luo X, Mitra D, Sullivan RJ, Wittner BS, Kimura AM, Pan S, et al Isolation and molecular characterization of circulating melanoma cells. Cell reports. 2014;7: 645–653. 10.1016/j.celrep.2014.03.039 24746818 PMC4079008

[16] Mocellin S, Hoon D, Ambrosi A, Nitti D, Rossi CR. The prognostic value of circulating tumor cells in patients with melanoma: A systematic review and meta-analysis. Clin Cancer Res. 2006;12: 4605–4613. 16899608 10.1158/1078-0432.CCR-06-0823

[17] Nicholl MB, Elashoff D, Takeuchi H, Morton DL, Hoon DS. Molecular upstaging based on paraffin-embedded sentinel lymph nodes: Ten-year follow-up confirms prognostic utility in melanoma patients. Ann Surg. 2011;253: 116–122. 10.1097/SLA.0b013e3181fca894 21135695 PMC3046555

[18] Hoshimoto S, Faries MB, Morton DL, Shingai T, Kuo C, Wang HJ, et al Assessment of prognostic circulating tumor cells in a phase III trial of adjuvant immunotherapy after complete resection of stage IV melanoma. Ann Surg. 2012;255: 357–362. 10.1097/SLA.0b013e3182380f56 22202581 PMC3320770

[19] Hoshimoto S, Shingai T, Morton DL, Kuo C, Faries MB, Chong K, et al Association between circulating tumor cells and prognosis in patients with stage III melanoma with sentinel lymph node metastasis in a phase III international multicenter trial. J Clin Oncol. 2012;30: 3819–3826. 10.1200/JCO.2011.40.0887 23008288 PMC3478576

[20] Scoggins CR, Ross MI, Reintgen DS, Noyes RD, Goydos JS, Beitsch PD, et al Prospective multi-institutional study of reverse transcriptase polymerase chain reaction for molecular staging of melanoma. J Clin Oncol. 2006;24: 2849–2857. 16782924 10.1200/JCO.2005.03.2342

[21] Shay JW, Bacchetti S. A survey of telomerase activity in human cancer. Eur J Cancer. 1997;33: 787–791. 9282118 10.1016/S0959-8049(97)00062-2

[22] Kim NW, Piatyszek MA, Prowse KR, Harley CB, West MD, Ho PL, et al Specific association of human telomerase activity with immortal cells and cancer. Science. 1994;266: 2011–2015. 7605428 10.1126/science.7605428

[23] Kojima T, Hashimoto Y, Watanabe Y, Kagawa S, Uno F, Kuroda S, et al A simple biological imaging system for detecting viable human circulating tumor cells. J Clin Invest. 2009;119: 3172–3181. 10.1172/JCI38609 19729837 PMC2752068

[24] Maida Y, Kyo S, Sakaguchi J, Mizumoto Y, Hashimoto M, Mori N, et al Diagnostic potential and limitation of imaging cancer cells in cytological samples using telomerase-specific replicative adenovirus. Int J Oncol. 2009;34: 1549–1556. 19424572 10.3892/ijo_00000284

[25] Ju M, Kao GD, Steinmetz D, Chandrasekaran S, Keefe SM, Guzzo TJ, et al Application of a telomerase-based circulating tumor cell (CTC) assay in bladder cancer patients receiving postoperative radiation therapy: A case study. Cancer Biol Ther. 2014;15: 683–687. 10.4161/cbt.28412 24618718 PMC4049784

[26] Macarthur KM, Kao GD, Chandrasekaran S, Alonso-Basanta M, Chapman C, Lustig RA, et al Detection of brain tumor cells in the peripheral blood by a telomerase promoter-based assay. Cancer Res. 2014;74: 2152–2159. 10.1158/0008-5472.CAN-13-0813 24525740 PMC4144786

[27] Dorsey JF, Kao GD, MacArthur KM, Ju M, Steinmetz D, Wileyto EP, et al Tracking viable circulating tumor cells (CTCs) in the peripheral blood of non—small cell lung cancer patients undergoing definitive radiation therapy: Pilot study results. Cancer. 2015;121: 139–149. 10.1002/cncr.28975 25241991 PMC4270850

[28] Kudo LC, Vi N, Ma Z, Fields T, Avliyakulov NK, Haykinson MJ, et al Novel cell and tissue acquisition system (CTAS): Microdissection of live and frozen brain tissues. PLoS One. 2012;7: e41564 10.1371/journal.pone.0041564 22855692 PMC3404047

[29] Zong C, Lu S, Chapman AR, Xie XS. Genome-wide detection of single-nucleotide and copy-number variations of a single human cell. Science. 2012;338: 1622–1626. 10.1126/science.1229164 23258894 PMC3600412

[30] Lin J, Goto Y, Murata H, Sakaizawa K, Uchiyama A, Saida T, et al Polyclonality of BRAF mutations in primary melanoma and the selection of mutant alleles during progression. Br J Cancer. 2011;104: 464–468. 10.1038/sj.bjc.6606072 21224857 PMC3049568

[31] Kang SY, Ahn S, Lee S, Jeong JY, Sung J, Oh YL, et al Shifted termination assay (STA) fragment analysis to detect BRAF V600 mutations in papillary thyroid carcinomas. Diagn Pathol. 2013;8: 121 10.1186/1746-1596-8-121 23883275 PMC3751688

[32] Cleves MA, Rock L. From the help desk: Comparing areas under receiver operating characteristic curves from two or more probit or logit models. The Stata Journal. 2002;2: 301–313.

[33] Long GV, Menzies AM, Nagrial AM, Haydu LE, Hamilton AL, Mann GJ, et al Prognostic and clinicopathologic associations of oncogenic BRAF in metastatic melanoma. J Clin Oncol. 2011;29: 1239–1246. 10.1200/JCO.2010.32.4327 21343559

[34] Chapman PB, Hauschild A, Robert C, Haanen JB, Ascierto P, Larkin J, et al Improved survival with vemurafenib in melanoma with BRAF V600E mutation. N Engl J Med. 2011;364: 2507–2516. 10.1056/NEJMoa1103782 21639808 PMC3549296

[35] Chiu CG, Nakamura Y, Chong KK, Huang SK, Kawas NP, Triche T, et al Genome-wide characterization of circulating tumor cells identifies novel prognostic genomic alterations in systemic melanoma metastasis. Clin Chem. 2014;60: 873–885. 10.1373/clinchem.2013.213611 24718909

[36] Heaphy CM, Subhawong AP, Hong SM, Goggins MG, Montgomery EA, Gabrielson E, et al Prevalence of the alternative lengthening of telomeres telomere maintenance mechanism in human cancer subtypes. Am J Pathol. 2011;179: 1608–1615. 10.1016/j.ajpath.2011.06.018 21888887 PMC3181356

[37] Tsai J, Lee JT, Wang W, Zhang J, Cho H, Mamo S, et al Discovery of a selective inhibitor of oncogenic B-raf kinase with potent antimelanoma activity. Proc Natl Acad Sci U S A. 2008;105: 3041–3046. 10.1073/pnas.0711741105 18287029 PMC2268581

[38] Yu X, Ambrosini G, Roszik J, Eterovic AK, Stempke-Hale K, Seftor EA, et al Genetic analysis of the 'uveal melanoma' C918 cell line reveals atypical BRAF and common KRAS mutations and single tandem repeat profile identical to the cutaneous melanoma C8161 cell line. Pigment Cell Melanoma Res. 2014. 10.1111/pcmr.12345 25515650

