# Supplementary figures and images for: A Novel Approach for the Detection and Genetic Analysis of Live Melanoma Circulating Tumor Cells
Source: PLoS One. 2015 Mar 25;10(3):e0123376. doi: 10.1371/journal.pone.0123376 (PMC4373770; doi:10.1371/journal.pone.0123376)

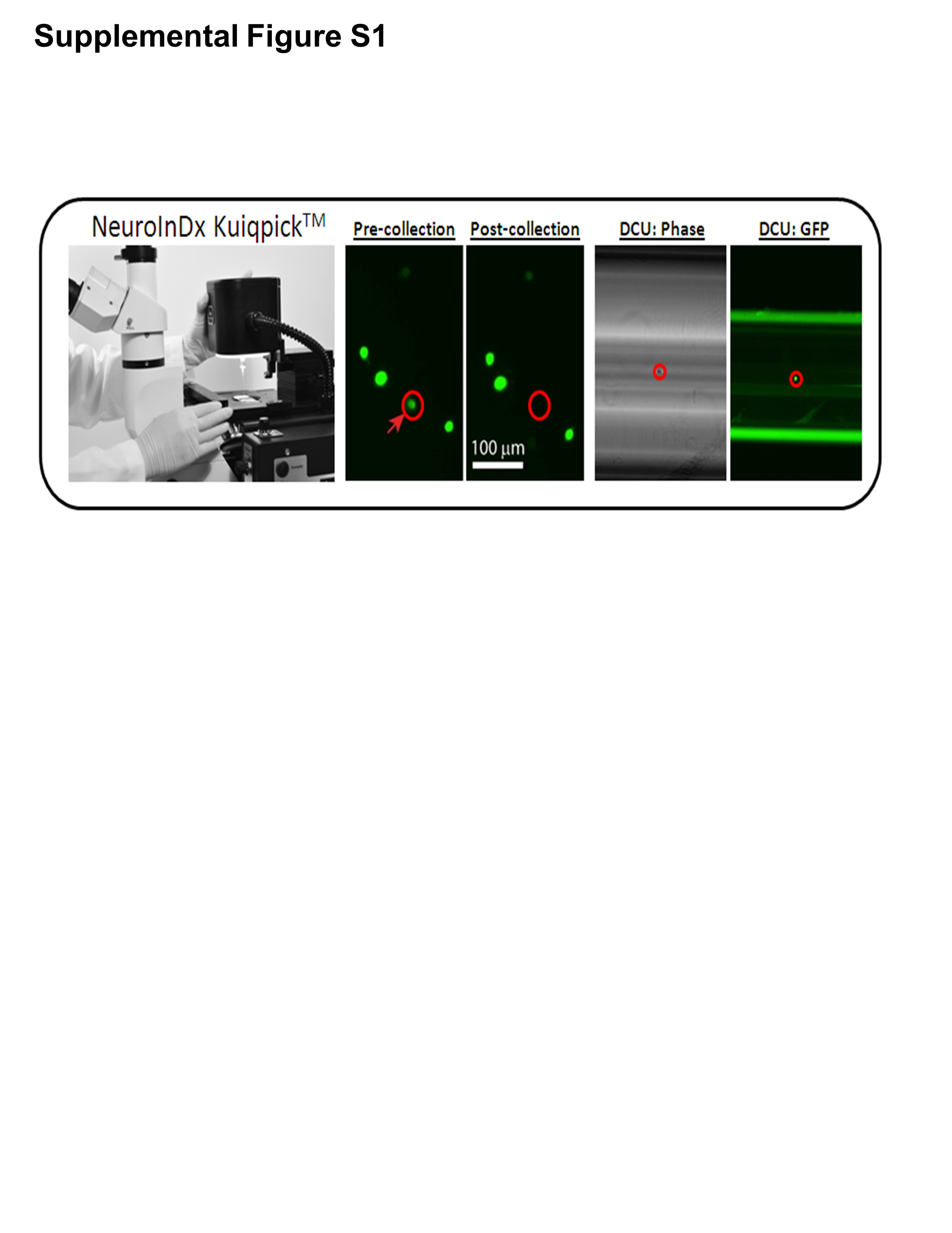

Supplement: S1 Fig — In vitro validation of capillary-based isolation of GFP probe-expressing cancer cells. Pre-collection and post-collection imaging of a target cell expressing GFP that is removed by capillary-based isolation (red circle). Disposable capillary unit (DCU) imaging (under phase contrast and fluorescence (GFP) conditions) demonstrates the capture and isolation of a single cell. (TIF) [file pone.0123376.s001.tif]

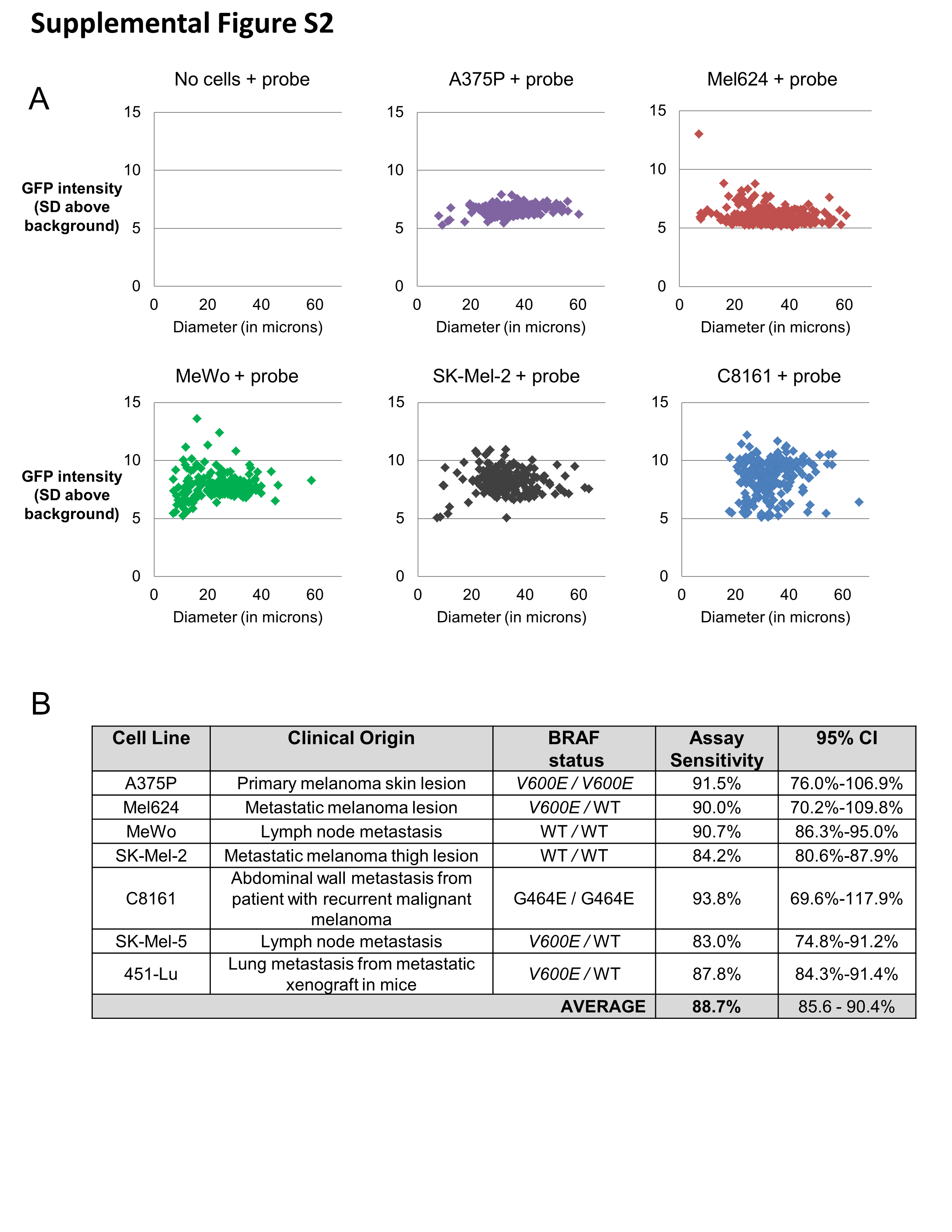

Supplement: S2 Fig — (A) Representative scatter plots showing individual cells, plotting size (X-axis) and fluorescent intensity (standard deviations (SD) above background) (Y-axis) identified by the imaging program. Seven melanoma cell lines were incubated with the probe and GFP+ cells were enumerated using size and fluorescence intensity criteria described in Methods. Hoechst staining was used to detect the number of nuclei in the well. Data for five cell lines shown: A375P (380 GFP+/428 nuclei), Mel624 (400 GFP+/417 nuclei), MeWo (256 GFP+/267 nuclei). SK-Mel-2 (307 GFP+/348 nuclei), and C8161 (271 GFP+/287 nuclei). Colors are for aesthetic purposes only. (B) Table describing each cell line’s clinical origin, BRAF mutation status, and sensitivity to the probe with 95% CI. Sensitivity calculated by dividing # GFP+ cells by # Hoechst-stained nuclei. 95% CI calculated by 4–6 repeated experiments for each cell line. The C8161 melanoma cell line is BRAF G464E mutant and wild type at the 600 codon [37,38]. (TIF) [file pone.0123376.s002.tif]

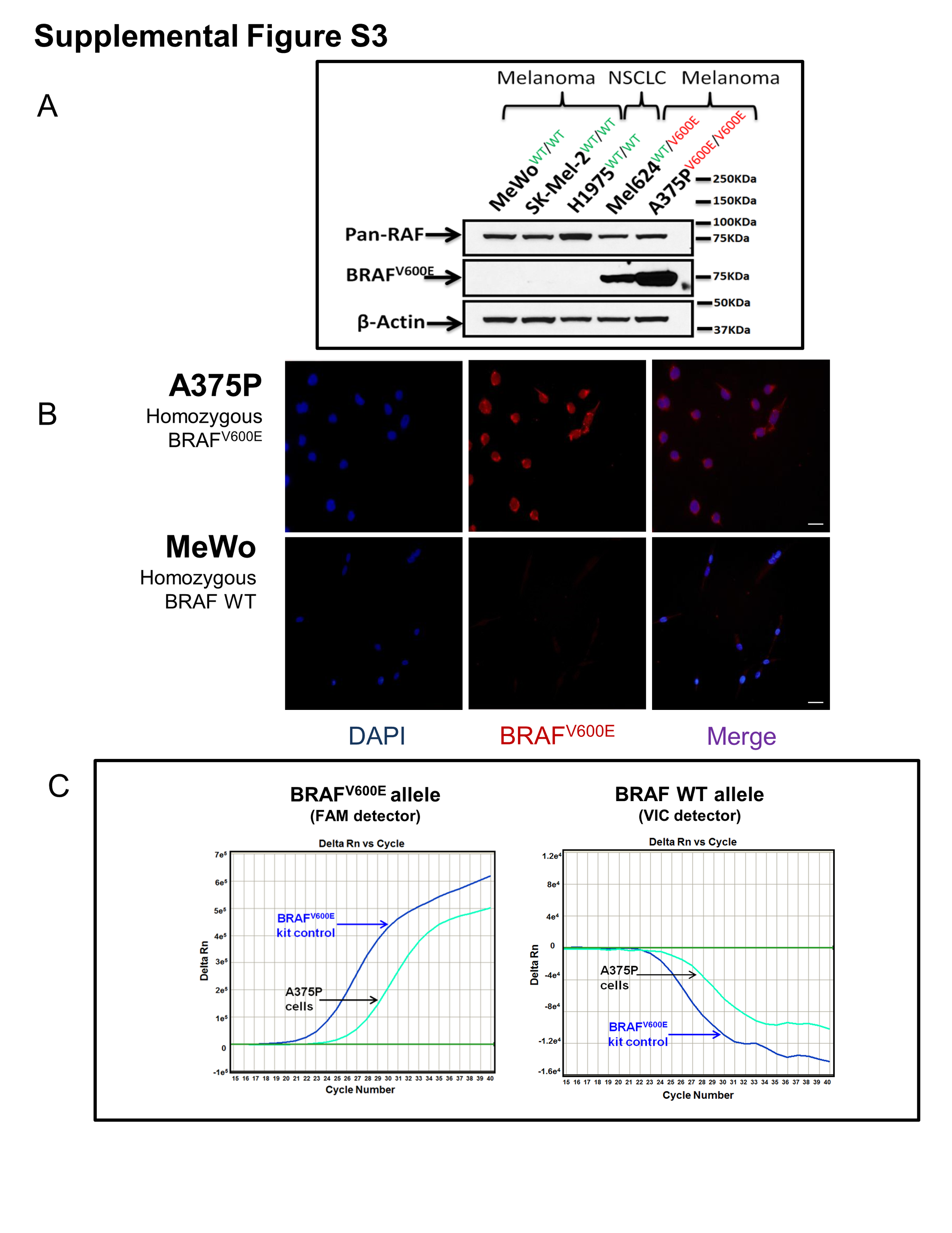

Supplement: S3 Fig — (A) Western blotting showing that BRAF protein (“Pan-RAF”, top blot) is present in all cell lines. However, probing with an antibody specific for the mutated BRAF protein (“BRAFV600E”, middle blot) reveal that only A375P and Mel624 express the mutated protein. This data is consistent with sequencing results for each cell line as well as the subsequent WGA and qPCR analysis. Probing for β-actin served as a loading control. NSCLC = non-small cell lung cancer. (B) Immunofluorescence staining of A375P (homozygous BRAFV600E) and MeWo (homozygous BRAF WT) cell lines with DAPI and the BRAFV600E antibody, are consistent with the western blot and sequencing results. Bar, 30 um. (C) The A375P cell line was incubated with the probe and DNA was extracted, amplified, and subject to qPCR analysis for the BRAF allele. PCR results demonstrated amplification of the BRAFV600E allele and absence of amplification of the BRAF WT allele. (TIF) [file pone.0123376.s003.tif]

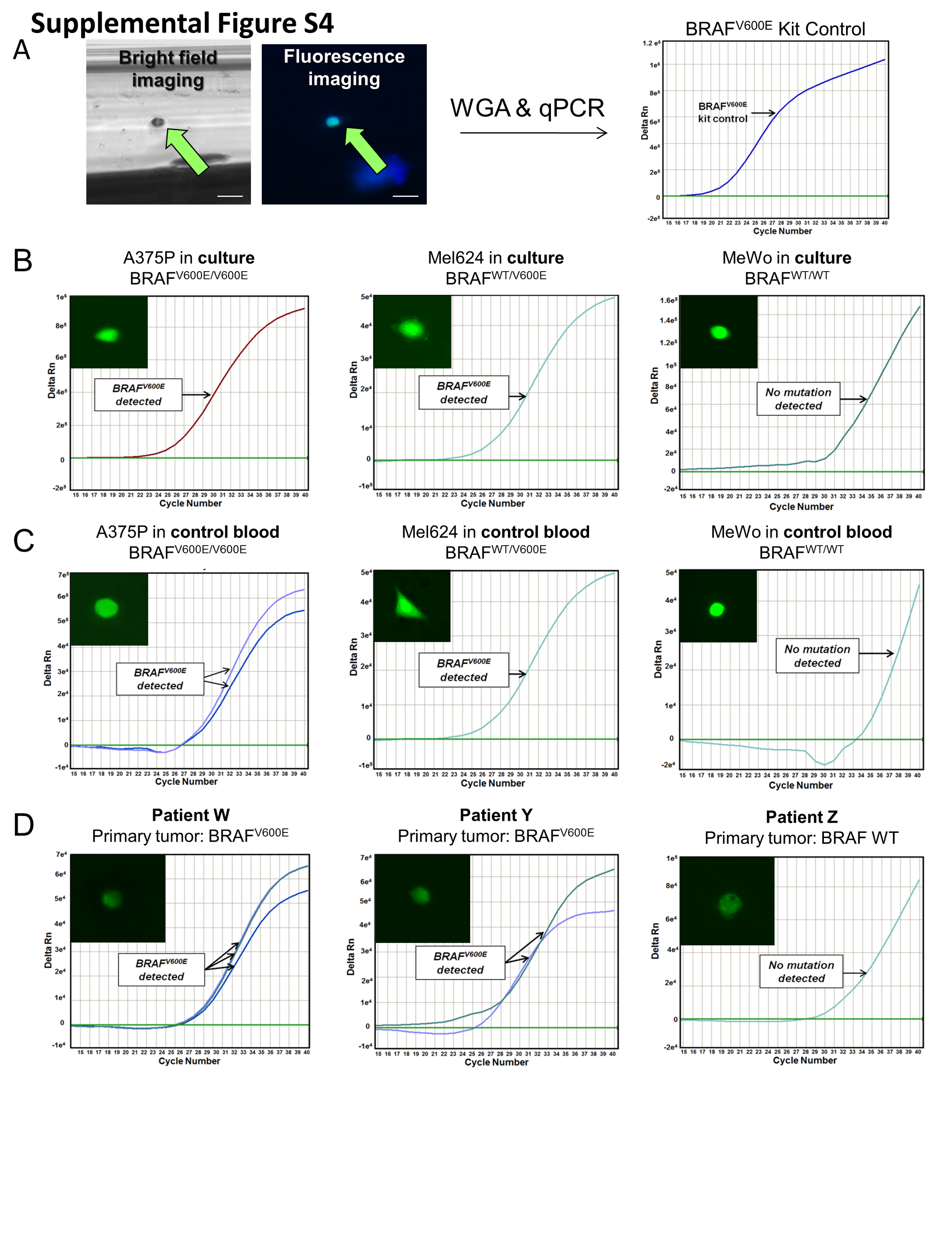

Supplement: S4 Fig — (A) Isolation, processing, and analysis of individual cells. Cells exposed to the probe and rendered fluorescent were isolated via capillary-based methods. The individual cells within the glass capillary tubes can be visualized under bright field (left) and fluorescence microscopy (right). Whole genome amplification (WGA) was performed on the DNA extracted from each cell, followed by quantitative polymerase chain reaction (qPCR) analysis using primers specific for the BRAFV600E mutation. The presence of the mutation results in signal (Delta Rn, Y-axis) detectable by the 28th cycle and a curve of the characteristic shape (as shown in the graph resulting from the BRAFV600E kit control). Bar, 30 um. (B) Isolation and genetic analysis of melanoma cells in culture. A375P (homozygous BRAFV600E), Mel624 (heterozygous BRAFV600E), and MeWo (homozygous BRAF WT) cells were isolated using the capillary-based technique described. The DNA was extracted from each cell and subject to WGA, followed by qPCR analysis with primers specific for the BRAFV600E mutation. Inset images show representative isolated cells. In each case, the qPCR analysis confirms the specific BRAF status of the parental cells in culture. (C) Isolation and genetic analysis of melanoma cells spiked into control blood. Melanoma cells were prepared as in (B) but spiked into blood from healthy volunteers. The subsequent isolation, DNA extraction, WGA, and qPCR analysis for BRAF mutations was not impeded by the presence of blood, and again the results matched that of the original cells. (D) Isolation of CTCs from patients and subsequent genetic analysis for BRAF mutation status. These methods described above were was applied to blood samples from an additional cohort of patients, with CTCs isolated via capillary-based methods followed by DNA extraction, WGA, and qPCR analysis for BRAF. In each case, the BRAF mutation status of the isolated CTC corresponded to that of the primary tumor. qPCR amplification curves demo [file pone.0123376.s004.tif]

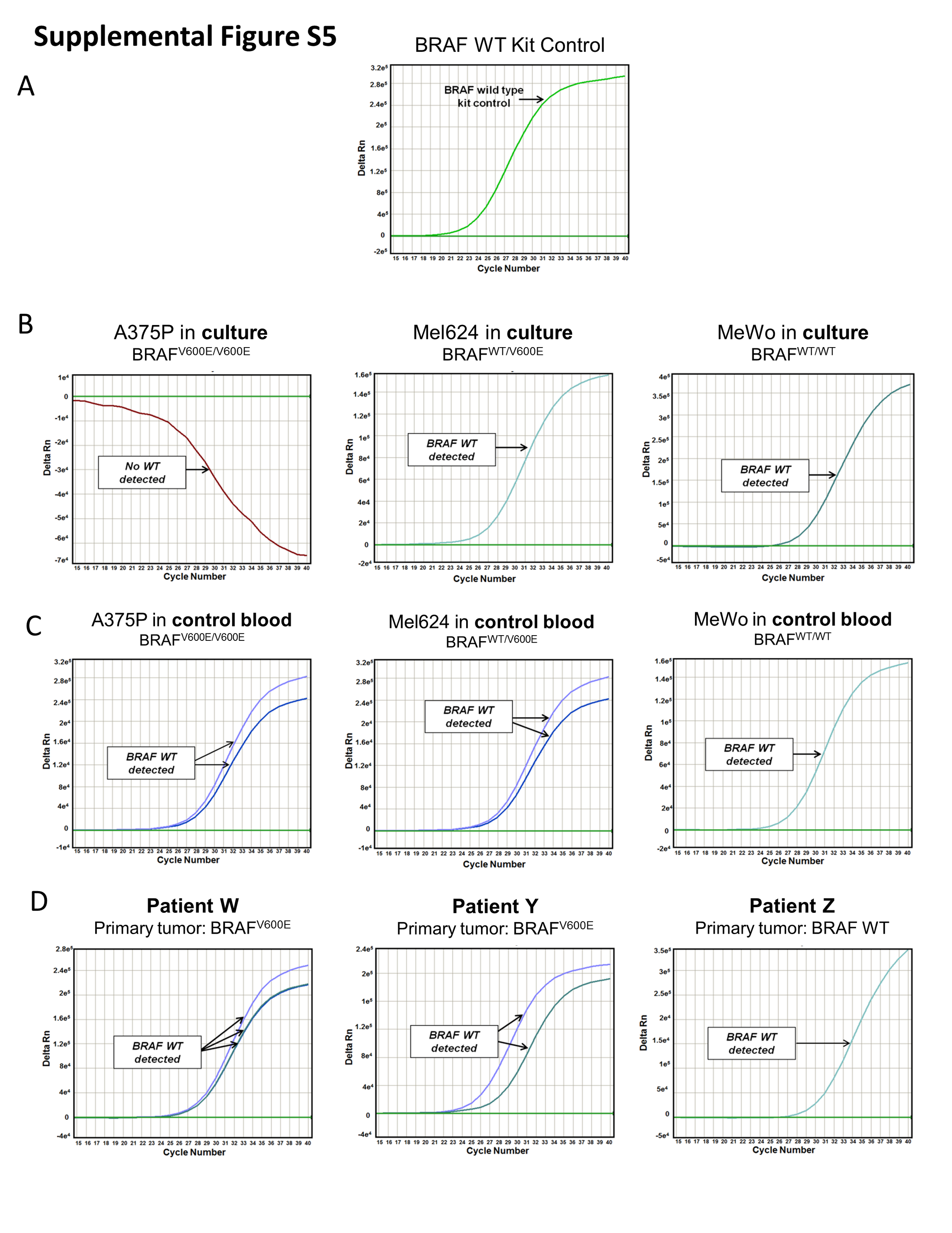

Supplement: S5 Fig — Each isolated melanoma cell analyzed for the BRAFV600E mutation qPCR analysis also underwent qPCR analysis using primers specific for BRAF WT. (A) The presence of the WT allele results in signal (Delta Rn, Y-axis) detectable by the 28th cycle (as shown in the graph resulting from the BRAF WT kit control). (B) A375P (homozygous BRAFV600E), Mel624 (heterozygous BRAFV600E), and MeWo (homozygous BRAF WT) cells were exposed to the probe, followed by capillary-based isolation, WGA, and qPCR analysis. In each case, the results of the qPCR analysis confirm the specific BRAF allele status of the parental cells in culture. (C) Isolation and genetic analysis of melanoma cells spiked into control blood. Melanoma cells were prepared as in (B) but spiked into blood from healthy volunteers. The qPCR analysis demonstrated non-melanoma-specific BRAF WT amplification in each case. This is likely a result of WBCs co-isolating with cancer cells under the capillary-based cell collection protocol. (D) Isolation of CTCs from patients and subsequent genetic analysis for BRAF mutation status. The qPCR analysis also demonstrated non-melanoma-specific BRAF WT amplification in each case. As in the case of melanoma cells spiked into control blood, the presence of BRAF WT DNA is likely due to normal WBCs co-isolating along with the cancer cells, and thus the WBC DNA also being co-amplified during WGA. (TIF) [file pone.0123376.s005.tif]
